# The effect of nonpharmacological interventions on pain and sleep quality after percutaneous nephrolithotomy: A protocol for systematic review and network meta-analysis

**DOI:** 10.1097/MD.0000000000028898

**Published:** 2022-03-11

**Authors:** Shibao Fu, Zhibo Mo, Shuming He, Xianping Che, Tingming Wu

**Affiliations:** Department of Urology Surgery, The Second Affiliated Hospital of Hainan Medical University, Haikou, Hainan province, China.

**Keywords:** network meta-analysis, nonpharmacological interventions, pain, percutaneous nephrolithotomy, protocol, sleep

## Abstract

**Background:**

Various nonpharmacological interventions have been applied to alleviate pain and improve sleep quality after percutaneous nephrolithotomy. However, evidence to compare their efficacy is scant. This study aims to evaluate the efficacy of different nonpharmacological interventions on alleviating pain and improving sleep quality in patients after percutaneous nephrolithotomy through a network meta-analysis.

**Methods:**

Randomized controlled trials reporting the efficacy of nonpharmacological interventions on alleviating pain and improving sleep quality in patients after percutaneous nephrolithotomy will be searched in online databases, including the Chinese Scientific Journal Database, China National Knowledge Infrastructure Database, Wanfang, China Biomedical Literature Database, Pubmed, Web of Science, Embase, and Cochrane Library. After quality assessment and date extraction, network meta-analysis will be performed using Stata 14.0 and R software.

**Results:**

The results of this meta-analysis will be submitted to a peer-reviewed journal for publication.

**Conclusions:**

This study will provide systematic and comprehensive evidence-based support for the effects of nonpharmacological interventions on alleviating pain and improving sleep quality after percutaneous nephrolithotomy.

**Ethics and dissemination:**

Ethical approval was not required for this study. The systematic review will be published in a peer-reviewed journal, presented at conferences, and shared on social media platforms.

**REGISTRATION NUMBER:**

DOI 10.17605/OSF.IO/B4DHW.

## 1 Introduction

Percutaneous nephrolithotomy is a minimally invasive treatment of kidney stones and some upper ureteral stones.^[1,2]^ It is featured by less bleeding, fewer complications, and higher stone removal rate.^[3,4]^ However, patients experience postoperative pain at varying degrees, which not only increases physical and psychological pain and affects sleep quality, but also causes ventilatory dysfunction, agitation, and even the risk of bleeding.^[5]^ These uncomfortable symptoms significantly influence the early postoperative recovery.^[6]^

For postoperative pain control and sleep improvement, pharmacological interventions have been applied,^[7,8]^ including opioid analgesics, nonopioid analgesics, benzodiazepines, etc.^[9]^ The use of analgesic, sedative, hypnotic, and antipsychostatic drugs, however, results in adverse events.^[10]^ Therefore, it is important to explore nonpharmacological interventions to control pain and improve sleep quality after percutaneous nephrolithotomy. Compared with pharmacological interventions, nonpharmacological interventions are low-cost, low-risk, and simple to perform, which have been widely used for various diseases.^[11–14]^

Although many randomized controlled trials (RCTs) have reported the effects of nonpharmacologic interventions on alleviating pain and improving sleep quality after percutaneous nephrolithotomy, their results may be inconsistent or even contradictory.^[5,15–17]^ In addition, evidence for comparing the effectiveness of these strategies is scant, which may affect the clinical application and scientific reliability of the results.

Compared with traditional meta-analysis, network meta-analysis (NMA) analyzes both direct and indirect evidence that provides quantitative statistical data of different interventions, and allows ranking the superiority of outcome indicators, thus providing evidence-based support for clinical practice. In this study, we will perform NMA to compare the effects of different nonpharmacological interventions on alleviating pain and improving sleep quality after percutaneous nephrolithotomy, thus providing systematic and comprehensive evidence-based support for clinical practice.

## 2 Methods

### 2.1 Study registration

This study has been registered in the OSF Registries (registration number: DOI 10.17605/OSF.IO/B4DHW), which follows the statement guidelines of preferred reporting items for systematic reviews and meta-analyses protocol.^[18]^

### 2.2 Eligibility criteria of inclusion of studies

#### 2.2.1 Types of studies

RCTs reporting the application of nonpharmacological interventions on alleviating pain and improving sleep quality after percutaneous nephrolithotomy.

#### 2.2.2 Types of participants

Patients older than 18 years after percutaneous nephrolithotomy.

#### 2.2.3 Types of interventions

Nonpharmacological interventions defined as any treatment without a proven or supposed pharmacological strategy, including aromatherapy, acupuncture, auricular therapy, massage, progressive muscle relaxation training, mindfulness therapy, etc. Conventional care measures or other types of interventions are applied in control group.

#### 2.2.4 Types of outcome measures

Postoperative pain and sleep quality scores measured with standardized instruments.

### 2.3 Exclusion criteria

1. Republications;
2. Studies with incomplete data;
3. Studies with inconsistent outcomes.

### 2.4 Data sources

The following electronic databases will be used to search for relevant RCTs using MeSH terms and free words: Chinese Scientific Journal Database, China National Knowledge Infrastructure Database, Wanfang, China Biomedical Literature Database, Pubmed, Web of Science, Embase, and Cochrane Library. The retrieval time of electronic database will be limited to January 2022.

### 2.5 Searching strategy

The detailed searching strategy of PubMed was given in Table 1, and literature search in other online databases will be similarly conducted.

**Table 1:** Search strategy in PubMed database.

Number	Search terms
#1	Nephrostomy, Percutaneous[MeSH]
#2	Nephrolithotomy, Percutaneous[Title/Abstract]
#3	Percutaneous Nephrolithotomy[Title/Abstract]
#4	Percutaneous Nephrostomy[Title/Abstract]
#5	Nephrolithotomies, Percutaneous[Title/Abstract]
#6	Nephrostomies, Percutaneous[Title/Abstract]
#7	Percutaneous Nephrolithotomies[Title/Abstract]
#8	Percutaneous Nephrostomies[Title/Abstract]
#9	or/1–8
#10	Pain[MeSH]
#11	Suffering, Physical[Title/Abstract]
#12	Ache[Title/Abstract]
#13	Pain, Burning[Title/Abstract]
#14	Pain, Crushing[Title/Abstract]
#15	Pain, Migratory[Title/Abstract]
#16	Pain, Radiating[Title/Abstract]
#17	Pain, Splitting[Title/Abstract]
#18	Aches[Title/Abstract]
#19	Burning Pain[Title/Abstract]
#20	Burning Pains[Title/Abstract]
#21	Crushing Pain[Title/Abstract]
#22	Crushing Pains[Title/Abstract]
#23	Migratory Pain[Title/Abstract]
#24	Migratory Pains[Title/Abstract]
#25	Pains, Burning[Title/Abstract]
#26	Pains, Crushing[Title/Abstract]
#27	Pains, Migratory[Title/Abstract]
#28	Pains, Radiating[Title/Abstract]
#29	Pains, Splitting[Title/Abstract]
#30	Physical Suffering[Title/Abstract]
#31	Physical Sufferings[Title/Abstract]
#32	Radiating Pain[Title/Abstract]
#33	Radiating Pains[Title/Abstract]
#34	Splitting Pain[Title/Abstract]
#35	Splitting Pains[Title/Abstract]
#36	Sufferings, Physical[Title/Abstract]
#37	Sleep[MeSH]
#38	Sleep, Slow-Wave[Title/Abstract]
#39	Sleep, Slow Wave[Title/Abstract]
#40	Slow-Wave Sleep[Title/Abstract]
#41	or/10-40
#42	Randomized Controlled Trial[MeSH]
#43	Random∗[Title/Abstract]
#44	Clinic trial [Title/Abstract]
#45	or/42-44
#46	#10 and #41 and #45

### 2.6 Data collection and analysis

#### 2.6.1 Literature screening and data extraction

**Figure 1:**
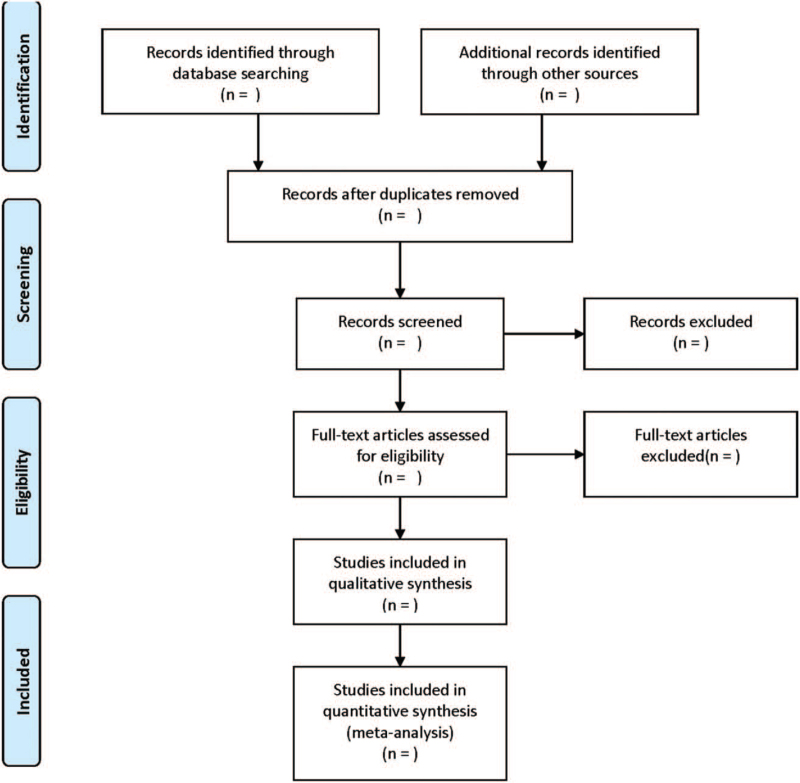
Flow diagram of study selection process.

PRISMA flow diagram will be used to summarize the results of the whole selection process (Fig. 1). Two evaluators will be responsible for literature screening, data extraction, and cross-check independently. Any disagreement will be solved by the third evaluator. In literature screening, the title and abstract will be read first. After excluding obviously irrelevant literatures, the full-text will be further reviewed. The following data will be extracted and filled into the preestablished data extraction table: general information of literatures (eg, author, date of publication, title), baseline characteristics of subjects, sample size, intervention measures, outcomes, and assessment of bias risk.

#### 2.6.2 Assessment of evidence quality

The risk of RCT bias will be evaluated by 2 evaluators according to the risk of RCT bias assessment tool in the Cochrane handbook.^[19]^

#### 2.6.3 Measures of outcomes

Continuous variables will be combined using the standardized mean differences and corresponding 95% confidence intervals.

#### 2.6.4 Management of missing data

Missing data will be requested by emailing the first author; otherwise, the data will be excluded from the study.

### 2.7 Data synthesis

#### 2.7.1 Pairwise meta-analysis

The heterogeneity will be assessed by the I^2^ test. I^2^≤50% considered as a small heterogeneity, and a fixed-effect model will be used to combine the effect size; otherwise, a random-effects model will be introduced.

#### 2.7.2 Consistency analysis

The consistency of direct comparison and indirect comparison will be judged by node splitting analysis in cases with closed rings. *P* > .05 suggests no significant difference between the direct and indirect comparison, and a consistency model will be used; otherwise, the inconsistent model will be adopted.

#### 2.7.3 Network meta-analysis

Stata14.0 software will be used for direct comparison, and network evidence plots will be drawn. R 4.1.2 software will be used for NMA, and the Bayesian method will be used to present the possibility of each intervention by ranking probability.

#### 2.7.4 Assessment of publication biases

A comparison-adjusted funnel plot will be depicted to identify the small sample effect between studies to assess the publication bias.^[20]^

#### 2.7.5 Subgroup analysis

Subgroup analysis will be performed based on the intervention time.

#### 2.7.6 Sensitivity analysis

The sensitivity analysis will be performed by eliminating 1 study at each time and calculating the remaining data.

#### 2.7.7 Ethics and dissemination

This study will not involve ethical approval or review and it will be presented in print or at relevant conferences.

## 3 Discussion

Postoperative pain after percutaneous nephrolithotomy should be well concerned.^[21,22]^ It not only affects sleep quality and increases physical and psychological pain, but also causes ventilatory dysfunction, agitation, and even the risk of bleeding.^[5]^ Opioids are the commonly used analgesic medications after percutaneous nephrolithotomy. However, they may cause adverse events, including nausea, decreased bowel movements, and sedation.^[7]^ Currently, nonpharmacological interventions have been highlighted, which have been applied in various fields. However, the effects of nonpharmacological interventions on alleviating pain and improving sleep quality after percutaneous nephrolithotomy are controversial. This study will conduct a NMA to directly and indirectly compare the effects of different nonpharmacological interventions on alleviating pain and improving sleep quality after percutaneous nephrolithotomy, thus providing evidence-based medical support for selecting the optimal postoperative interventions for percutaneous nephrolithotomy.

## Author contributions

**Conceptualization:** Tingming Wu, Shibao Fu.

**Data curation:** Shibao Fu, Zhibo Mo and Shuming He.

**Formal analysis:** Zhibo Mo.

**Funding acquisition:** Tingming Wu.

**Investigation:** Zhibo Mo.

**Methodology:** Zhibo Mo.

**Project administration:** Tingming Wu.

**Resources:** Zhibo Mo, Shuming He.

**Software:** Shuming He, Xianping Che.

**Supervision:** Tingming Wu.

**Validation:** Shuming He, Xianping Che.

**Visualization and software:** Zhibo Mo and Shuming He.

**Visualization:** Shuming He, Xianping Che.

**Writing – original draft:** Shibao Fu and Tingming Wu.

**Writing – review & editing:** Shibao Fu and Tingming Wu.
